# E151 (*sym15*), a pleiotropic mutant of pea (*Pisum sativum* L.), displays low nodule number, enhanced mycorrhizae, delayed lateral root emergence, and high root cytokinin levels

**DOI:** 10.1093/jxb/erv201

**Published:** 2015-05-06

**Authors:** James M. C. Jones, Lindsey Clairmont, Emily S. Macdonald, Catherine A. Weiner, R. J. Neil Emery, Frédérique C. Guinel

**Affiliations:** ^1^Biology Department, 75 University Avenue W, Wilfrid Laurier University, Waterloo, ON, Canada, N2L 3C5; ^2^Biology Department, 1600 West Bank Drive, Trent University, Peterborough, ON, Canada, K9J 7B8

**Keywords:** Abscisic acid, cytokinin, epidermal–cortex interface, infection thread, legume, mycorrhiza formation, nodulation, pea mutant, root development.

## Abstract

In legumes, the formation of rhizobial and mycorrhizal root symbioses is a highly regulated process which requires close communication between plant and microorganism. Plant mutants that have difficulties establishing symbioses are valuable tools for unravelling the mechanisms by which these symbioses are formed and regulated. Here E151, a mutant of *Pisum sativum* cv. Sparkle, was examined to characterize its root growth and symbiotic defects. The symbioses in terms of colonization intensity, functionality of micro-symbionts, and organ dominance were compared between the mutant and wild type. The endogenous cytokinin (CK) and abscisic acid (ABA) levels and the effect of the exogenous application of these two hormones were determined. E151 was found to be a low and delayed nodulator, exhibiting defects in both the epidermal and cortical programmes though a few mature and functional nodules develop. Mycorrhizal colonization of E151 was intensified, although the fungal functionality was impaired. Furthermore, E151 displayed an altered lateral root (LR) phenotype compared with that of the wild type whereby LR emergence is initially delayed but eventually overcome. No differences in ABA levels were found between the mutant and the wild type, but non-inoculated E151 exhibited significantly high CK levels. It is hypothesized that CK plays an essential role in differentially mediating the entry of the two micro-symbionts into the cortex; whereas it would inhibit the entry of the rhizobia in that tissue, it would promote that of the fungus. E151 is a developmental mutant which may prove to be a useful tool in further understanding the role of hormones in the regulation of beneficial root symbioses.

## Highlight

The phenotype of the E151 pea mutant, unique among known legume mutants, provides the first evidence for a promoting role for cytokinins in the development of the mycorrhizal symbiosis.

## Introduction

Many recent reviews have highlighted the intricacy of nodule development and the existence of the common signal transduction pathway involved in both the rhizobial and mycorrhizal mutualistic relationships (Bapaume and Reinhardt, 2012; Oldroyd, 2013). This pathway was unravelled in large part from the detailed study of nodulation mutants. Of particular interest are pleiotropic mutants because their phenotype is likely to be regulated by one (or more) hormone(s) (Karlowski and Hirsch, 2003). Their careful study may therefore point to the action of a specific hormone in nodule organogenesis or mycorrhiza formation. For example, R50 (*Pssym16*) is a pleiotropic mutant of *Pisum sativum* (Guinel and Sloetjes 2000), the study of which has highlighted a link between cytokinin (CK) and nodulation (Ferguson *et al.*, 2005) before a molecular connection was firmly established in *Medicago truncatula* by Gonzalez-Rizzo *et al.* (2006) and in *Lotus japonicus* by Murray *et al.* (2007). E151 (*Pssym15*) is a mutant that was created by mutagenesis of *P. sativum* cv. Sparkle seeds with ethylmethane sulphonic acid (Kneen *et al.*, 1994). It was characterized as having short and thin lower lateral roots (LRs), a short third internode, and rare nodulation. Via genetic analysis, Kneen *et al.* (1994) concluded that the abnormal nodulation was caused by a mutation in a single recessive gene which was named *sym15* and mapped to chromosome 7. The gene location was further refined to the linkage group VII of the pea genome (JIC Germplasm Collection, http://data.jic.ac.uk/cgi-bin/pgene/, accessed 22 April 2015), and yet the gene product and its role in regulating nodulation remained elusive.

In this study, the nodulation and mycorrhizal phenotypes of E151 were compared with those of the wild type (WT) to explore whether SYM15 is involved in the control of nodulation and mycorrhiza formation. In order to do so, nodule organogenesis and functioning, mycorrhiza formation and fungal symbiotic efficiency, and parameters characteristic of the root system were assessed or measured. Because E151 nodulation traits were reminiscent of those observed in the nodulation mutants *Mtcre1* of *M. truncatula* and *Ljhk1* of *L. japonicus* defective in perception of CK (Gonzalez-Rizzo *et al.*, 2006; Murray *et al.*, 2007, respectively), and because mycorrhizal E151 plants share several traits with mycorrhizal tomato plants treated with abscisic acid (ABA), it was hypothesized that E151 has higher levels of both CK and ABA. To test this hypothesis, the endogenous levels of the two hormones were measured in non-inoculated and inoculated plants. WT plants were also treated exogenously with ABA and the CK 6-benzylaminopurine (BAP) to determine whether the E151 LR phenotype could be mimicked.

## Materials and methods

### Plant and micro-symbiont growth conditions

Seeds from the WT Sparkle and the mutant E151 (*Pssym15*) were surface-sterilized in an 8% bleach solution, rinsed in sterile water, and left to imbibe for 18h. Seeds were placed in different substrates depending on the study undertaken as detailed below. All plants were grown in a growth room under a 16/8h, 23/18 ºC, light/dark regime, with 210 μEm^–2^ s^–1^ of photosynthetically active radiation provided by high pressure sodium and metal halide lamps. When required, non-symbiotic plants were watered, through a cycle initiated 10 days after planting (DAP) of nutrient solution (NS; Guinel and Sloetjes, 2000), water, water, fertilizer (N:P:K, 17:5:19), water, water, NS, etc. Plants inoculated with rhizobia and those with mycorrhizal fungi were subjected to a similar watering regimen; however, low-nitrogen NS [i.e. as above but with only 0.5mM Ca(NO_3_)_2_] and low-phosphate NS (i.e. as above but with only 0.4mM KH_2_PO_4_) were used, respectively. Furthermore, symbiotic plants never received fertilizer.

For the nodulation studies, unless otherwise stated, plants were inoculated 5 DAP with 5% yeast-mannitol broth culture (5ml) of *Rhizobium leguminosarum* bv. *viciae* strain 128C53K (gift of Dr Stewart Smith, EMD Crop BioScience, Milwaukee, WI, USA). For the mycorrhizal studies, plants were grown in the presence of the arbuscular mycorrhizal fungus *Rhizophagus irregulare* (DAOM 197918), obtained originally from the Agriculture and Agri-Food Canada Glomeromycota *in vitro* collection (AAFC, Ottawa, ON, Canada), but now propagated in the lab on leek (*Allium ampeloprasum*) roots grown in a mixture (1:1, v:v) of Turface^®^:peat [Plant Products Company Ltd (Brampton, ON, Canada) and ASB Greenworld Ltd (Mount Elgin, ON, Canada), respectively]. The inoculum was prepared by harvesting the soil with colonized leek roots, cutting the roots into small pieces, and later mixing soil and roots with the substrate in a 1:6 (v:v) ratio.

### E151 nodulation phenotype

Plants were grown in a sterile combination (1:1, v:v) of Turface^®^: vermiculite (Plant Products Company Ltd). Control plants did not receive any inoculant. At 14, 21, 28, 35, and 42 days after inoculation (DAI), the nodule number and nodule dry weight (DW) per plant were recorded. Any bump visibly protruding from the root with a single narrow attachment point was considered to be a nodule.

To study nodule organogenesis, seedlings were transferred to growth pouches (Mega International, West St. Paul, MN, USA) 3 DAP as per Clemow *et al.* (2011). Plants in pouches were placed in pots filled with sterilized substrate and received the same watering regimen as other rhizobia-inoculated plants. At 8 DAP, five LRs which exhibited an elongation zone with root hairs susceptible to rhizobial infection were spot-inoculated per plant, and the location of the event was marked on the outer side of the pouch. Each root received 0.5 μl of a 5% rhizobial solution (Clemow *et al.*, 2011). Three plants of each line were randomly harvested at 3, 7, 10, 14, 21, 28, and 35 DAI. From these, three of the five spot-inoculated LRs were cut 0.5cm on either side of the mark where the bacteria were placed. The LR segments were fixed under vacuum [1h; 1.25% (v/v) glutaraldehyde solution (Marivac, Canton de Gore, QC, Canada) made with 0.1M phosphate buffer (pH 7.0)], cleared under vacuum (6min; 30% bleach), and vacuum-infiltrated with glycerol (1h in 30% and 1h in 60%). The unstained segments were observed with a light microscope equipped with phase-contrast optics. Nodulation events were scored and the number of nodulation events per LR segment (=1cm) was determined. The root segments were observed with a Nikon Eclipse 50i compound microscope and photographs were taken with a Pax-Cam Arc digital camera and Pax-it 7.0 imaging software (www.paxit.com).

To determine which organ, the root or the shoot, controls the E151 nodulation phenotype, reciprocal grafts (WT/E151 and E151/WT) and isografts (WT/WT and E151/E151) were achieved according to Resendes *et al.* (2001) with some modifications. Seeds were planted in a mix (1:1, v:v) of Turface^®^:vermiculite and, to facilitate grafting, germinating seedlings were kept in the dark for 4 d. Etiolated plants were grafted and placed back in the growth room in a humid chamber until they were inoculated 5 d later. The number of nodules on their stock was counted 21 DAI.

To estimate nitrogenase activity, seeds were planted in growth pots (Qubit Systems, Kingston, ON, Canada) filled with sterilized grade 16 silica sand (Bell and Mackenzie, Hamilton, ON, Canada) which was wetted the night before the seeds were planted. Because at the time of measurements the pots had to be covered with a lid, the seeds were planted just beneath the surface and covered only with a thin layer of sand. The pots were placed in trays filled with water or NS, according to the watering regimen, and kept in the growth room. Plants were not watered for 2 d prior to plant measurements to allow for the substrate to dry. Seedlings were inoculated 3 DAP with *R. leguminosarum* bv. *viciae* strain 128C79 (gift of Dr Stewart Smith) which lacked the uptake hydrogenase enzyme (Nelson and Salminen, 1982). Nitrogenase activity was estimated by measuring hydrogen evolution (Hunt and Layzell, 1993) with a Qubit apparatus and Logger Pro 3 software which was calibrated according to the manufacturer’s manual. Plants (16 WT and 14 E151) were assessed once a week, from 14 DAI to 42 DAI; the amount of hydrogen evolved from the nodulated plants was measured using the open flow gas exchange system (Sulieman and Schulze, 2010; Macdonald, 2011).

To determine nitrogen and ethylene sensitivity of the plants, individual seeds were planted in a mixture (1:1, v:v) of Turface^®^:vermiculite, and seedlings were inoculated with rhizobia. Seedlings were subjected either to nitrogen (modified from Bloom *et al.*, 2003) or to ethylene treatments. For the former, 5 DAP and every 3 d thereafter, plants received 100ml of either KNO_3_ (8, 10, or 12mM) or NH_4_Cl (2, 5, or 8mM) added to a nitrogen-free NS with 2.5mM CaNO_3_ replaced by 2mM CaSO_4_ and applied as described above. For the latter, plants received at the root crown, 7 DAP and every 3 d thereafter, 5ml of either Ag_2_SO_4_ (1 μM or 10 μM), 1-aminocyclopropane-1-carboxylic acid (ACC; 10 μM or 20 μM), or amino-vinyl-glycine (AVG; 10 μM or 20 μM) dissolved in water. For the nitrogen experiment, all plants were given water as needed between treatment applications, whereas for the ethylene experiment, plants received low-nitrogen NS once a week. For both experiments, the control treatment consisted of the low-nitrogen NS mentioned above. At 35 DAI, the nodule number and DWs of the root system and nodules were determined.

### E151 mycorrhizal phenotype

Seeds were planted individually in a sterile mixture (1:1, v:v) of Turface^®^:peat, to which fungal inoculum had been added. Plants were harvested 35 DAI and three randomly selected LRs were excised from the primary root (PR). These roots were cut into sections 3cm in length, cleared with 10% KOH, stained with a 5% ink–vinegar solution, de-stained in 10% vinegar solution, and stored in 50% glycerol (modified from Vierheilig *et al.*, 1998). Processed roots were mounted in 50% glycerol and scanned along their entire length using a microscope. The numbers of hyphopodia, arbuscules, and vesicles formed per colonization unit were recorded. From these numbers, the colonization units per centimetre of LR (i.e. infection units; Franson and Bethlenfalvay, 1989) and the percentage cortical entry were calculated.

To assess which organ controls the E151 mycorrhizal phenotype, reciprocal grafts and isografts were performed. Seeds were planted in a combination (1:1, v:v) of Turface^®^:peat, and germinating seedlings were subjected to etiolation for 4 d. On the day of grafting, seedlings were transferred to a substrate containing the fungal inoculum. Immediately, their shoots were cut and exchanged to create reciprocal grafts and isografts; the grafted plants were then placed for 5 d in a humid chamber in the growth room. The assessment of mycorrhizae was performed 35 DAI.

To determine mycorrhizal efficiency, plants were grown in a mix (1:1, v:v) of Turface^®^:peat containing the fungal inoculum. Plants were harvested 35 DAI and all LRs located within 10cm of the cotyledons were separated from the PR and cut into 3cm segments. Of these segments, seven were randomly chosen for each stain. As a positive control, seven additional root segments were stained with ink–vinegar. Roots were stained and assessed for fungal alkaline phosphatase (ALP) activity (Tisserant *et al.*, 1993; Van Aarle *et al.*, 2002) and for accumulation of polyphosphates (PolyP; Ezawa *et al.*, 2003). After staining, the root segments were vacuum-infiltrated in 30% and then in 60% glycerol. Samples were mounted on slides in 60% glycerol. Each stained root segment was categorized as follows: for ALP, as fully stained (no unstained fungal structures seen), partially stained (some structures stained), or not stained (structures present but not stained); for PolyP, as heavy partial staining (some fungal structures intensely stained), light partial staining (some fungal structures with visible staining), or no staining (fungal structures visible but unstained). For both stains, the segments in each category were counted for each plant line, and that number was divided by the total number of colonized segments to give a percentage.

### E151 lateral root phenotype

Seeds were planted in Conetainers^®^ (Stuewe & Sons, Inc., Tangent, OR, USA) in a mix (1:1, v:v) of Turface^®^:vermiculite. Seedlings were harvested 3 DAP and every 3 d thereafter up to 15 DAP when PR length, number of emerged LRs, length of the longest LR, and the length of the LR-free zone (distance between the cotyledons and the first emerged LR) were measured. Plants of the same age but grown separately had their PR cleared as in Pepper *et al.* (2007) to assess their number of LR primordia.

In an attempt to phenocopy the E151 root system, seeds were planted as above. Seedlings were treated exogenously, 2 DAP and every 3 d thereafter, with a range of concentrations (0, 1, 5, 10, and 15 μM) of ABA (A1049 from Sigma-Aldrich, Oakville, ON, Canada) or BAP (13151-1G-F from Sigma-Aldrich). These two chemicals were dissolved in 0.01M NaOH. For the ABA treatments, WT and E151 seeds were used, whereas for the BAP treatments, only WT seeds were planted. Plants were harvested 6 and 12 DAP, and the same parameters as above were measured.

### Endogenous measurements of abscisic acid and cytokinin in E151

Nodulated and mycorrhizal plants, grown in the appropriate substrate, were harvested 6 DAI. Their roots were separated from the shoots, cleaned, and freeze-dried for several hours. The extraction and purification of both ABA and CK were performed following a modified protocol described by Quesnelle and Emery (2007) and Farrow and Emery (2012). Accordingly, samples were homogenized (ball mill, Retsch MM300) at 4 °C over ice (–20 °C) and spiked with internal standards including 149.8ng of ^2^H_4_ABA (Plant Biotechnology Institute, SK, Canada) and 10ng of each of ^2^H_7_BA, ^2^H_7_[9R]cBA, ^2^H_5_ZOG, ^2^H_3_DHZOG, ^2^H_5_[9R]ZOG, ^2^H_3_[9R]DHZOG, ^2^H_6_iP7G, ^2^H_5_Z9G, ^2^H_5_MeSZ, ^2^H_6_MeSiP, ^2^H_5_[9R]MeZ, ^2^H_5_[9R]MeSiP, ^2^H_6_[9R]iP, ^2^H_5_[9R]Z, ^2^H_3_[9R]DHZ, ^2^H_6_iP, ^2^H_3_DHZ, ^2^H_5_Z, ^2^H_6_iPMP, ^2^H_5_ZRMP, and ^2^H_3_DHZRMP (OlchemIm Ltd, Olomouc, Czech Republic). Extraction residues were reconstituted in 0.2ml of 1M formic acid (pH 1.4) and purified on a mixed mode, reverse-phase, cation-exchange cartridge (Oasis MCX 2cc; Waters, Mississauga, ON, Canada), resulting in separate fractions of ABA, CK nucleotides (CKNTs), CK free bases (CKFBs), and CK ribosides (CKRs). CKNTs were dephosphorylated (Emery *et al.*, 2000), converted to, and analysed as CKRs. Prior to liquid chromatography–tandem mass spectrometry (LC-MS/MS) analysis, all sample fractions were reconstituted in 1.5ml of buffer [CH_3_COOH:C_2_H_3_N:double-distilled H_2_O (0.08:5.0:94.92, v:v:v) for CK, and CH_3_COOH:CH_3_OH:ddH_2_O (0.08:5.0:94.92, v:v:v) for ABA].

To quantify and analyse the amounts of ABA and CK obtained, high-performance liquid chromatography-electrospray ionization tandem mass spectrometry (HPLC-ESI MS/MS) measurements [Agilent 1100 series HPLC connected to a Sciex Applied Biosystem 5500 API Mass spectrometer and with a turbo V-spray ionization source and multiple reaction monitoring (MRM) channels, one specific for each analyte] were carried out (Ross *et al.*, 2004; Farrow and Emery, 2012). Detection limits were as listed in Farrow and Emery (2012). A 20 μl sample was injected onto a Luna C18 reverse-phase column (3 μm, 150×2.0mm; Phenomenex, Torrance, CA, USA); ABA samples and all CK samples were analysed in negative-ion and positive-ion modes, respectively. HPLC conditions for ABA and CK were followed as per Noble *et al.* (2014). Data were analysed using the Analyst (v. 4.2.1) software (AB SCIEX, Concord, ON, Canada). ABA and CK were identified based on their MRM channels and retention times. Concentrations were determined according to an isotope dilution analysis based on direct comparison of the endogenous analyte peak area with that of the recovered internal standard. In cases where unlabelled standards were not available, analytes were identified based on their relative retention time with a corresponding deuterated standard. Final hormone concentrations were determined using the total weight of the sample analysed.

### Statistical analysis

Mean values and standard errors (SEs) were generated for all data sets; they were subjected to different statistical tests depending on the comparison performed. Student’s *t*-tests were used to compare data obtained from the spot inoculation, the mycorrhizal colonization, and the seedling establishment experiments. A non-parametric Mann–Whitney U-test was used to analyse the results from the grafting experiments (nodulation and mycorrhizae). One-way analyses of variance (ANOVAs) were performed followed by Fischer’s Protected LSD for the exogenous treatments with nitrogen and ethylene, by Tukey’s HSD for the exogenous treatments with ABA and BAP, and by Duncans’ test for the endogenous hormonal analysis. Tests were completed using either Sigma Plot 11.0 (Systat Software, Inc., San Jose, CA, USA), Statistica 6.0 (Statsoft, Tulsa, OK, USA), or R (R Core Team, 2013). For each experiment, the number of plants used per trial is indicated within the table footnotes and figure legends.

## Results

### E151 is a low and delayed nodulator with blocks in both the epidermal and the cortical programmes

**Fig. 1.**
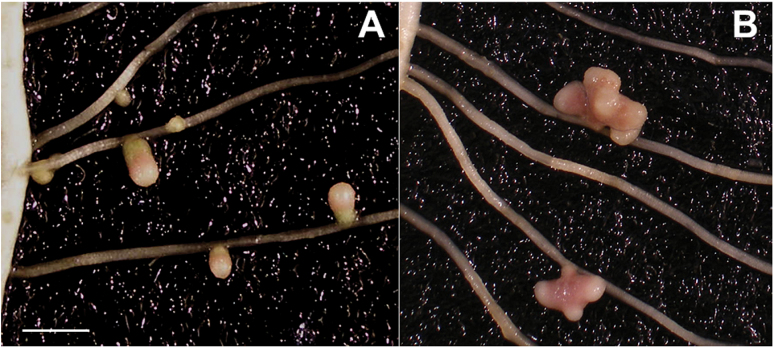
Nodules from the WT (A) and E151 (B) 28 days after inoculation. Whereas the WT exhibits small cylindrical nodules, E151 displays large, fan-shaped nodules which are multimeristematic. Scale bar=1cm. (This figure is available in colour at *JXB* online.)

The low nodulating phenotype of E151 (Kneen *et al.*, 1994) was confirmed in this study, with E151 displaying no more than 55 nodules and the WT as many as 210 at 28 DAI (Table 1). The lower nodule number did not reflect E151’s ability to interact with rhizobia because the mutant exhibited a number of infection events per centimetre of root segment similar to that of the WT, with an average of 4.1±3.2 and 3.9±2.6 for E151 and the WT, respectively. Here, E151 has been further characterized as a delayed nodulator (Table 1) because there was a 1 week delay between the WT and E151 for nodule appearance, number, and onset of pink coloration. E151 nodules were delayed in their formation and functioning, as noticed by their differences in coloration: WT nodules exhibited a green base earlier than E151 nodules, indicating earlier senescence. Mature E151 nodules were heavier than those of the WT (Table 1), probably because of their multilobed morphology (Fig. 1). E151 nodules exhibited up to five lobes when the WT nodules generally had a single lobe. Finally, E151 displayed an atypical nodulation pattern which has been reported in detail by Remmler *et al.* (2014).

**Table 1.** Nodulation parameters of the WT and E151 The plants were inoculated with *Rhizobium leguminosarum* bv. *viciae* and harvested over a period of 6 weeks.

	14 DAI		21 DAI		28 DAI		35 DAI		42 DAI	
	WT	E151	WT	E151	WT	E151	WT	E151	WT	E151
Nodule number	190.04±10.75	6.79±1.54 a	213.58±11.97	29.08±3.74 b	209.71±11.63	53.50±4.96 c	209.42±11.52	49.04±3.77	210.29±10.33	44.58±2.45
Total nodule DW^*a*^ (mg)	22.30±1.17 a	0.38±0.11 a	54.10±5.48 b	10.30±1.60 b	69.80±9.44 b	27.60±2.38 c	106.00±13.30 c	35.40±2.27 d	104.00±12.60 c	36.90±3.48 d
Individual nodule DW^*a*^ (mg)	0.121 ± 0.006 a	0.035±0.007 a	0.258±0.022 b	0.377±0.066 b	0.329±0.037 c	0.573±0.060 c	0.542±0.082 d	0.855±0.103 d	0.538±0.086 d	0.871±0.087 d

Data (mean ±SE; *n*=24 per line per time period over three trials) were subjected to Student’s *t*-tests (95% confidence level). All parameters were found to be significantly different between the two pea lines. The different letters show significant differences between consecutive plant ages within a pea line.

^*a*^ Only the nodules removed easily from the roots were taken into account; the small bumps not fully developed were not collected.

DAI, days after inoculation; DW, dry weight.

**Fig. 2.**
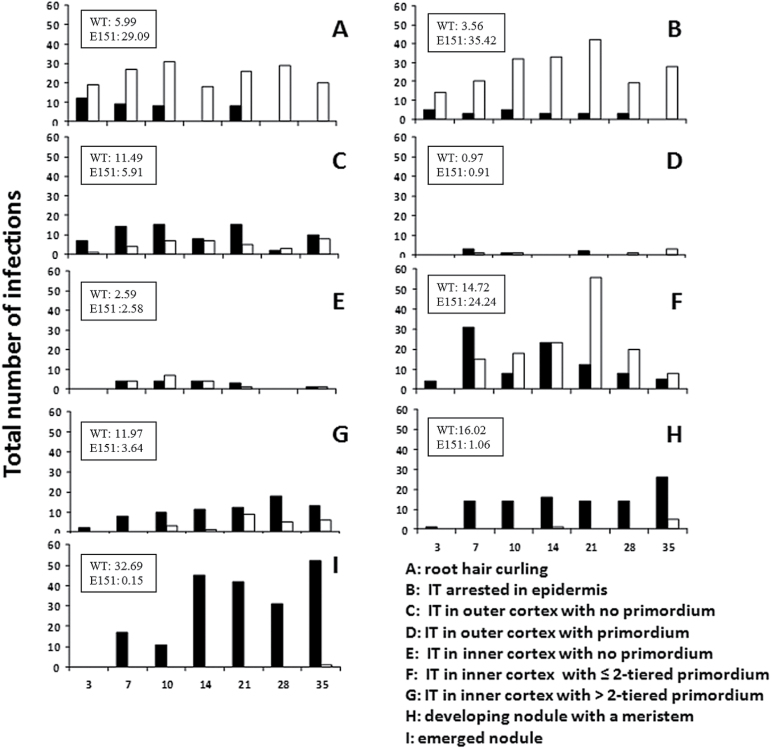
Total number of nodulation events for each stage of WT (black bars) and E151 (white bars) root segments at 3, 7, 10, 14, 21, 28, and 35 days after inoculation. Nine stages have been defined as A–I, and insets show percentages of infection events observed on lateral roots at each of those stages. The inset values represent all time points combined in relation to the total number of infections observed for each pea line (618 for the WT and 660 for the mutant E151). The total number of plants was at least 22 for each line and time.

**Fig. 3.**
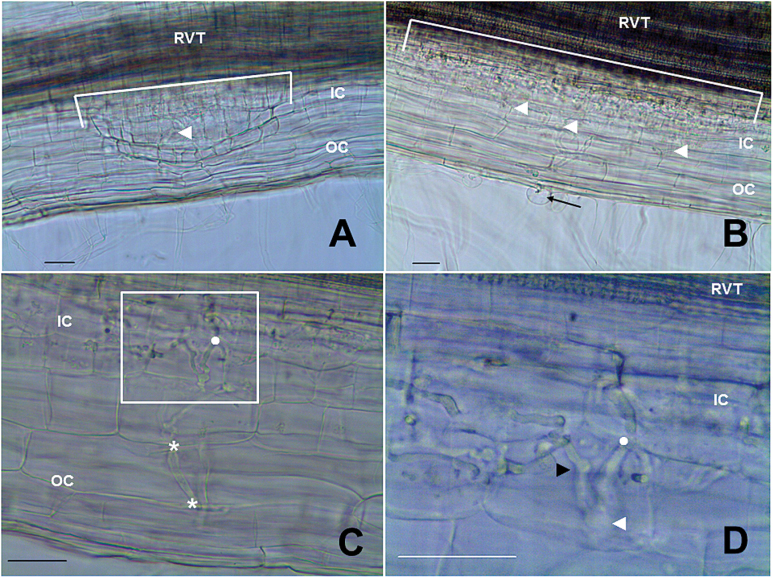
Nodule development on WT (A) and E151 (B–D) plants grown in pouches and spot-inoculated. Lateral roots were cleared but not stained. Nodule stages are those described in Fig. 2. (A) Nodule (stage H) with a meristem forcing its way through the outer cortex (OC). The nodule originates in the inner cortex (IC) from the cortical cells abutting the root vascular tissue (RVT). The nodule base delineated by the white line covers a length of 350 μm. An infection thread (arrowhead) is seen within the developing nodule. (B) Nodule primordium (stage F) comprised only of two cell layers and with a wide base [575 μm (white line)]. The infection thread starts from a curled root hair (arrow) and branches many times (arrowheads). (C) Close-up on an infection thread illustrating its splitting each time it enters into a new cell layer (*) of the OC. In the IC, the branching becomes erratic. The area highlighted by the box is magnified in (D) and the dot is located in the same place in both images. (D) The infection thread exhibits bulges (white arrowhead) and knots (black arrowhead) in the IC. Many branches of the thread enter into a single cell which can no longer divide; consequently, the IC cells become enlarged. Scale bars=50 μm. (This figure is available in colour at *JXB* online.)

To determine whether E151 was blocked in its nodule development, nodule organogenesis was followed over time, and the occurrence of specific nodulation stages was compared between the WT and mutant. Four points will be highlighted here. First, in E151 many more root hairs responded to bacterial inoculation than in the WT, and this responsiveness to rhizobia lasted much longer in E151 than in the WT (Fig. 2, stage A). Therefore, in E151, the zone of infection remained susceptible for a longer time period than in the WT. Second, E151 was delayed in all nodulation stages analysed (Fig. 2). Evidence for this was seen as early as 3 DAI when rare infection threads (ITs) had penetrated the cortex (Fig. 2, stage C), no IT could be linked to a young primordium in E151 (Fig. 2, stages F and G), and no developing nodule was observed (Fig. 2, stage H). Third, the ITs in E151 behaved atypically (compare Fig. 3B with Fig. 3A). They not only had difficulty breaching the epidermis–cortex interface, but they also ramified extensively when they reached the inner cortex. An IT often doubled its branches each time it entered a new cortical cell (Fig. 3C), progressing in many directions and becoming non-uniform in thickness (Fig. 3D). Fourth, there was an apparent loss of co-ordination between the epidermal and cortical nodulation programmes, with two apparent ‘blocks’ occurring during E151 nodule organogenesis (Fig. 2). In E151, nearly 30% of all ITs were observed in the root epidermis (Fig. 2, stage B) and almost 25% of all ITs were associated with dividing nodule progenitor cells (Fig. 2, stage F), when corresponding numbers in the WT were 4% and ~14%, respectively. In E151, ITs either stopped abruptly at, or grew parallel to, the inner periclinal walls of the epidermal cells. As for the nodule founder cells, those of E151 underwent only two rounds of cell divisions, dividing first anticlinally and then periclinally. Because the base of the primordium was much larger in E151 than in the WT, the number of anticlinal divisions occurring in the innermost cortical cell layer had to be higher (Fig. 3A, B). However, despite the defective signalling between the two programmes, some nodules were capable of full development and of nitrogen fixation (see below).

**Fig. 4.**
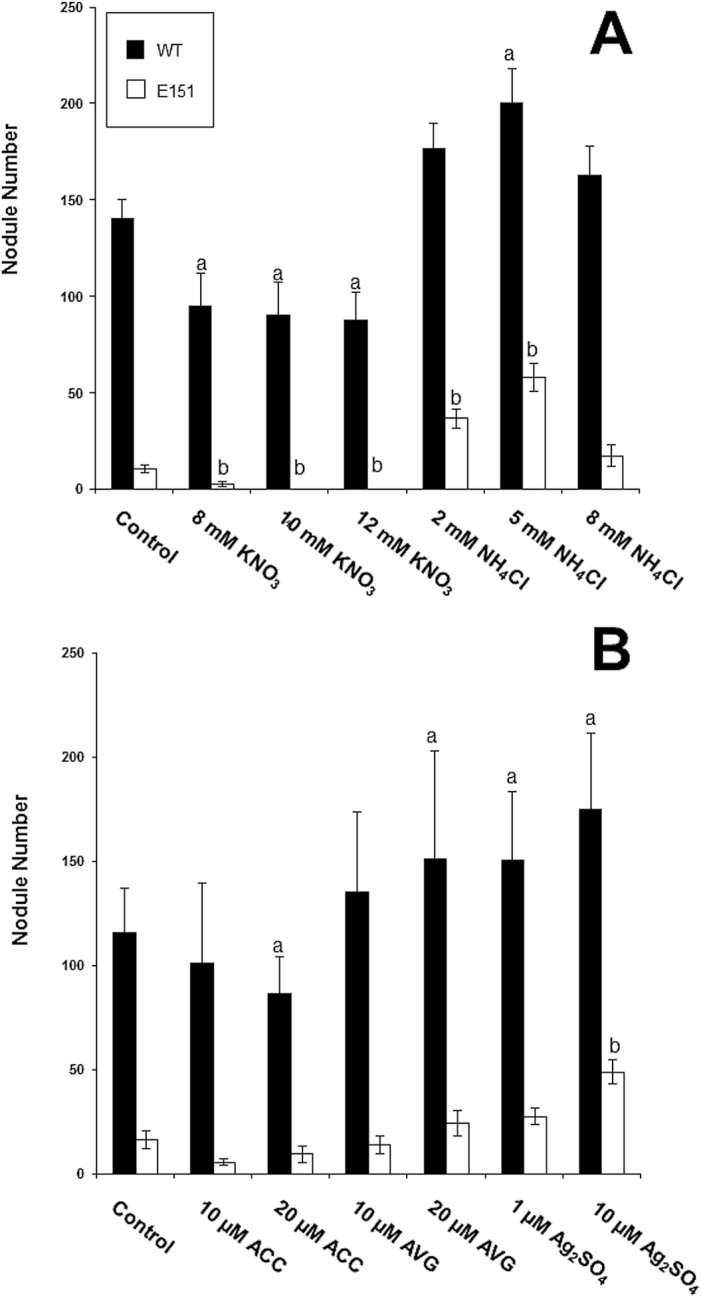
(A) Nodule number on WT and E151 plants treated with either nitrate or ammonium. (B) Nodule number on WT and E151 plants treated with either the direct precursor of ethylene (ACC), an inhibitor of the enzyme ACC synthase (AVG), or an antagonist of ethylene action (Ag_2_SO_4_). Data (mean ±SE; nitrogen treatments: *n*=12 per line, three trials; ethylene treatments: *n*=15 per line, four trials) were subjected to a one-way ANOVA followed by Fischer’s protected LSD post-hoc test to determine significant differences within pea lines between treatments; letters (a for WT and b for E151) indicate significance at a 95% confidence level.

To understand E151 nodulation further, a physiological approach was taken. Through reciprocal grafts, it was established that the E151 nodulation phenotype was root controlled. Whereas E151/WT (scion/stock) bore 150±12 nodules on its lateral roots, WT/E151 had only 53±10; these numbers should be compared with those of the isografts, namely 214±23 (WT/WT) and 47±7 (E151/E151). By measuring hydrogen evolution, it was determined that E151 nodules were capable of fixing nitrogen (N_2_), but later than the WT. On average, for the WT, N_2_ fixation was 2.0 μmol H_2_ h^–1^ at 21 and 28 DAI but declined by 35 DAI. In contrast, for E151, N_2_ fixation was apparent only after 28 DAI, at which time it increased significantly but only for a limited number of plants. At 35 DAI, only three of 14 plants fixed an amount larger than 1.5 μmol H_2_ h^–1^. To test the sensitivity of E151 nodules to inorganic nitrogen, both pea lines were treated with either nitrate or ammonium. Despite their nodule number difference, both pea lines responded to the nitrogen treatments similarly (Fig. 4A) and as expected (Fei and Vessey, 2009). Whereas nitrate significantly inhibited nodule formation, ammonium up to a concentration of 5mM promoted it. To determine ethylene sensitivity of E151, inoculated plants were treated with ACC, the ethylene precursor, AVG, an inhibitor of ACC synthase, or Ag_2_SO_4_, an antagonist of ethylene action. As expected in the WT (Guinel and Geil, 2002), when ACC or AVG was added to the substrate, nodule number was reduced or increased, respectively (Fig. 4B). Surprisingly, neither of these compounds significantly affected the mutant, though in both pea lines nodule formation was promoted by Ag_2_SO_4_ (Fig. 4B).

### E151 is a hypermycorrhizal mutant

**Fig. 5.**
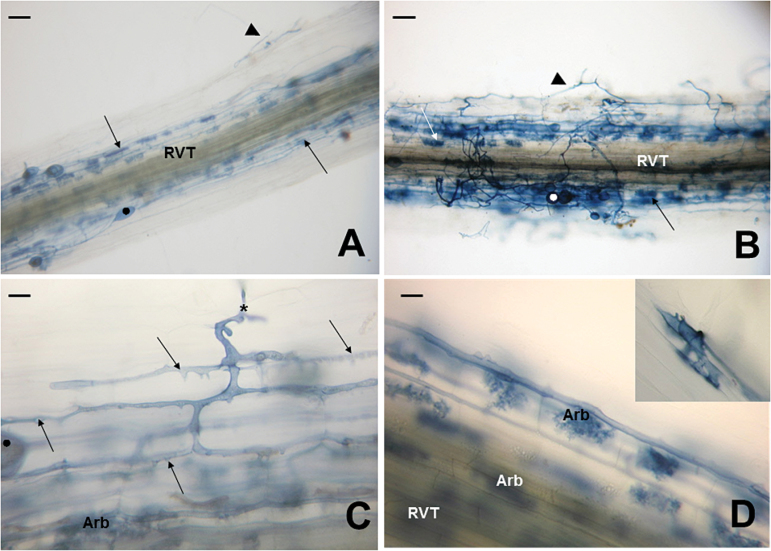
Mycorrhizae development in roots of WT (A) and E151 (B–D) plants inoculated with the fungus *Rhizophagus irregulare* and harvested 35 DAI. (A) Colonization unit exhibiting extraradicular hyphae (arrowhead), intraradical hyphae in the cortex running parallel to the root vascular tissue (RVT), many arbuscules (arrows), and vesicles (black dot). (B) Colonization unit comprising an abundance of extraradicular hyphae (arrowhead), arbuscules (arrow), and numerous vesicles. (C) Close-up of a hypha (asterisk) entering an epidermal cell and progressing through the root cortex. The hyphae run in the apoplastic space of the outer cortex, and, in contrast to those in the WT, which are smooth, they have short pointed projections (arrows) along their length. Most of the hyphae reaching the inner cortex develop into arbuscules (Arb). (D) The Arb are numerous as they are present in practically all of the inner cortical cells surrounding the stele (RVT). There are more hyphopodia in E151 than in the WT, and some are aberrant in that they are septate and branched (inset). Bars: (A) and (B)=200 μm; (C) and (D)=20 μm. (This figure is available in colour at *JXB* online.)

The AM fungus *R. irregulare* developed ~2.5 times more colonization units in E151 than in the WT (Table 2) and the units in E151 were more extensive than those in the WT (Fig. 5). The fungus on E151 differentiated twice as many hyphopodia as when growing on the WT (Table 2). About a third of these appeared atypical because they were larger than those on the WT, branched with multiple pegs, and were septate (inset, Fig. 5D). While not all deformed hyphopodia on E151 breached the root epidermis, those which did led to successful fungal spreading through the root cortex. Twice as many hyphae were able to progress into the E151 cortex as in the WT cortex (Table 2). In this tissue, the intraradicular hyphae branched extensively (Fig. 5C) in E151, giving rise to about four times more arbuscules and vesicles than in the WT (Table 2; Fig. 5D). The extensive branching is probably the cause of the larger colonization units in E151. Using reciprocal grafts, it was demonstrated that in E151, the hyphopodium number is controlled by both the root and the shoot, whereas the numbers of arbuscules and vesicles are regulated by the shoot only (Table 2).

**Table 2.** Quantitative parameters related to mycorrhizal phenotypes are given for the WT, E151, and reciprocal (WT/E151 and E151/WT) and non-reciprocal (WT/WT and E151/E151) grafts The plants were inoculated with *Rhizophagus irregulare* and harvested 35 days after inoculation.

	WT	WT/WT	WT/E151	E151/WT	E151/E151	E151
CU cm^–1^ root	0.13±0.03	0.08±0.02	0.13±0.04	0.17±0.05	0.21±0.05	0.36±0.09*
Hyphopodia per CU^†,‡^	1.74±0.31	1.45±0.16	1.84±0.17	1.96±0.21	2.51±0.21	3.27±0.28*
Cortex entry (%)	22.28±9.29	61.97±9.12	62.81±8.49	73.62±4.76	61.76±7.08	41.88±2.85*
Arbuscules per CU^†^	19.59±14.19	2.82±1.27	15.72±6.27	18.50±5.21	14.38±3.39	80.84±19.20*
Vesicles per CU^†^	2.49±2.21	0.25±0.17	1.29±0.69	1.79±0.65	1.55±0.42	9.67±6.81*

Data (mean ±SE; *n*=12 per line over three trials) were subjected to Student’s *t*-test between non-grafted pea lines and non-parametric Mann–Whitney U-test between grafted plants (95% confidence level).

An asterisk denotes a significant difference between non-grafted pea lines, whereas the symbol ‘†’ denotes a trait affected by the shoot only and the symbol ‘‡’ a trait affected by the root and the shoot. Cortex entry is given by the number of hyphae penetrating the cortex divided by the number of hyphopodia.

CU, colonization unit.

To assess whether the extensive mycorrhizal infection of E151 was efficient in terms of mutualism, the fungal ALP activity and the amount of fungal PolyP were estimated in mycorrhizal roots. WT roots harboured fungi which exhibited both highly active ALP in the intraradicular hyphae, arbuscules, and vesicles, and low accumulation of PolyP in the vesicles (Table 3). In E151 roots, the ALP activity was lower and patchy, with portions of the fungus unstained; also, the PolyP accumulation was occurring at irregular intervals as if it was scattered (Table 3).

**Table 3.** Functionality of the mycorrhizal fungus in the WT and E151 The plants were inoculated with *Rhizophagus irregulare* and harvested 35 days after inoculation. Roots were assessed for fungal alkaline phosphatase activity (ALP) and for accumulation of polyphosphates (PolyP). Root segments were assigned to a group based on their staining pattern.

	WT		E151	
	ALP	PolyP	ALP	PolyP
Fully stained/heavy partial (%)	97.1	18.2	14.6	23.8
Partially stained/light partial (%)	2.9	45.5	85.4	28.6
Unstained (%)	0.0	36.4	0.0	47.6
No visible colonization (%)	63.4	80.4	65.3	66.7

The data (*n*=14 plants ALP, *n*=8 plants PolyP, over two trials) are presented as the percentage of root segments falling into each staining group, calculated by dividing the number of root segments in that category by the total number of colonized roots. The percentage of non-colonized segments, obtained by dividing the number of non-colonized segments by the total number of segments analysed, is also given.

### Young E151 has a low number of emerged lateral roots but a large number of lateral root primordia

At 21 DAP, E151 has been described as having a shorter PR and three times fewer emerged LRs than the WT (Kneen *et al.*, 1994). Here E151 root architecture was further studied with a focus on seedling establishment. No differences were found between the PRs of the two pea lines; however, there were noticeable differences in their LRs (Table 4). Six DAP, emerged LRs of E151 were fewer and shorter than those of the WT (Table 4) and located further down on the PR axis. The first emerged LR of WT was found 0.68±0.08cm below the cotyledons whereas that of E151 was 1.24±0.08cm below (Table 5). Furthermore, for the first 12 d, the number of E151 LR primordia, which were not morphologically altered, was twice that of the WT (Table 4). E151 was evidently capable of overcoming the early handicap as by 15 DAP its emerged LR number exceeded that of the WT and its LR primordia number was similar. However, throughout development, E151 LRs remained shorter than those of the WT (Table 4).

**Table 4.** Growth parameters of non-inoculated WT and E151 throughout seedling establishment

	3 DAP		6 DAP		12 DAP		15 DAP	
	WT	E151	WT	E151	WT	E151	WT	E151
No. of emerged LRs	8.25±2.42	1.17±0.77*	28.42±2.00	18.58±2.49*	56.18±3.32	70.91±4.39*	46.91±5.43	69.36±4.40*
Longest LR length (cm)	0.52±0.12	0.37±0.12	3.88±0.20	2.28±0.33*	16.53±0.40	13.48±0.74*	18.78±0.63	15.61±0.53*
LR primordia number	12.67±1.10	28.75±5.26*	12.92±2.76	43.75±5.17*	12.82±1.93	23.91±3.79*	14.82±3.32	18.73±2.61
LR primordia density	1.49±0.13	3.12±0.47*	1.07±0.20	3.29±0.35*	0.59±0.09	1.29±0.22*	0.69±0.17	0.90±0.12
Emerged LR density	1.07±0.33	0.25±0.20*	2.80±0.36	1.41±0.18*	2.53±0.17	3.80±0.27*	2.33±0.24	3.32±0.18*

The data (mean ±SE; *n*=11–12, over three trials) were subjected to Student’s *t*-test (95% confidence level).

An asterisk indicates a significant difference between pea lines at the various time points.

Lateral root densities are given per centimetre of primary root.

DAP, days after planting; LR, lateral root.

**Table 5.** Root parameters of the WT treated with ABA compared with those of the non-treated E151 mutant Seedlings were not inoculated and were harvested 6 d after planting.

	WT				E151
ABA concentration (μM)	0	1	5	10	0
No. of emerged LRs	48.20±2.95	39.89±2.20	36±3.06	23.62±4.92 a	41.60±3.96
Longest LR length (cm)	4.61±0.23*	4.48±0.34	3.76±0.33	2.40±0.30 a	2.73±0.12*
LR-free zone (cm)	0.68±0.08*	1.26±0.12 a	1.37±0.14 a	1.55±0.22 a	1.24±0.08*
LR primordia number	23.00±2.25*	34.00±4.60	34.22±3.32	34.00±3.92	54.85±3.97*
LR primordia density	1.31±0.17*	1.87±0.25	2.02±0.21	2.25±0.28	3.49±0.26*
Emerged LR density	2.53±0.12	2.19±0.13	2.11±0.18	1.37±0.24 a	2.56±0.20

The data (mean ±SE; *n*=8–12 per line per treatment and *n*=20–22 for controls over three trials) were subjected to a one-way ANOVA followed by Tukey’s HSD post-hoc test (95% confidence level). An asterisk indicates a significant difference between pea lines, whereas the letter ‘a’ indicates a significant difference between control and ABA-treated WT plants.

Densities are given per centimetre of primary root.

At 6 DAP, E151 responded to ABA in a manner similar to the WT; however, at 12 DAP, the ABA effects were stronger (data not shown), suggesting that E151 older plants are more sensitive to ABA than WT plants. Of interest is that at 6 DAP, the root system of ABA-treated WT plants began to resemble that of untreated E151 (Table 5). The number and length of emerged LRs decreased and shortened, respectively; furthermore, the length of the LR-free zone as well as the number of LR primordia increased (Table 5). Thus it appears that ABA-treated WT phenocopies E151 in terms of LRs. As regards the cytokinin BAP, although it is known to have an inhibitory effect on LR formation and growth (Lorteau *et al.*, 2001), it did not appear to affect significantly WT plants (data not shown). Aside from the extended LR-free zone with increasing BAP concentrations, no other measured values were comparable with those of E151.

### The high levels of cytokinins found in E151 decreased upon symbiotic inoculation

The levels of the two hormones CK and ABA were measured 6 DAI in nodulated and mycorrhizal roots, as well as in control roots. The most interesting data concerned the CK levels (Table 6). First, non-inoculated (nod^–^ and myc^–^) E151 roots have significantly higher total CK levels than non-inoculated WT roots, especially when grown in Turface^®^:peat. Indeed, in that substrate, significant differences (*P*>0.05) were detected between the two lines for CKNTs, CKRs, and total CKs, with all CK forms higher in E151 than in the WT. For both pea lines, the CK precursors (CKNTs) were predominant, which is not surprising as a young root would be the primary site of CK synthesis. Second, whereas CK levels did not change upon rhizobial or fungal inoculation of WT plants, they decreased in E151 (*P*<0.05). The decrease was slight and not significant in the presence of rhizobia but strong and significant (*P*<0.05) in the presence of fungi (Table 6). With both symbionts, E151 roots had levels of CKNTs close to those of WT roots. Also, E151 potentially active CK (CKR and CKFB) levels decreased to levels close to those in the WT. This decrease of CK levels upon inoculation suggests that in the mutant the micro-symbionts were having an effect on the endogenous CK levels. As for ABA levels, these did not differ between roots of the two pea lines whether inoculated or not. However, these levels were affected by the substrates in which the plants grew. At 6 DAI, the roots of both WT and E151 plants grown in Turface^®^:vermiculite had twice as much ABA as those grown in Turface^®^:peat (Table 6).

**Table 6.** *Endogenous CK and ABA levels in roots of the WT and E151 inoculated with* Rhizobium leguminosarum bv. viciae (nod^+^), Rhizophagus irregulare (myc^+^), *or non-inoculated (*nod^–^ *and* myc^–^, *respectively)*. Rhizobia- and fungi-inoculated plants were grown on different substrates for 6 d after inoculation. Whereas the former grew in a mixture of Turface^®^:vermiculite, the latter grew in a mixture of Turface®:peat.

	CKNT	CKR	CKFB	Total CK	ABA
WT nod^–^	753.04±131.32	53.05±11.36	21.96±16.91	860.23±148.25	288.59±54.70
E151 nod^–^	909.26±193.44	70.09±14.76	43.37±27.32	1066.24±205.28	234.50±30.14
WT nod^+^	694.51±127.97	54.91±8.42	79.47±67.34	876.52±192.98	268.87±44.64
E151 nod^+^	693.89±77.26	53.93±6.24	14.24±6.97	826.05±96.79	338.71±35.06
WT myc^–^	670.67±93.70	85.65±13.86	54.36±33.53	853.45±117.27	148.07±26.53
E151 myc^–^	1271.84±270.07*	191.06±35.05*	183.79±108.62	1714.78±408.95*	128.33±18.92
WT myc^+^	740.82±152.77	85.23±19.10	26.68±15.42	935.02±179.22	123.09±24.56
E151 myc^+^	513.49±77.84 a	76.83±12.23 ^a^	10.72±6.20 a	661.50±97.61 a	105.68±14.30

All data are given in pmol g^–1^ of fresh weight. Nucleotides (CKNT) are CK precursors, whereas free bases (CKFB) and ribosides (CKR) are active CK forms.

Data (mean ±SE; *n*=4) were subjected to a one-way ANOVA followed by Duncan’s post-hoc test (95% confidence level). An asterisk indicates a significant difference between pea lines, whereas the letter ‘a’ indicates a significant difference between treatments within a pea line (myc^+^ versus myc^–^ and nod^+^ versus nod^–^).

## Discussion

### E151 is a unique mutant with a complex age-dependent phenotype

The most apparent phenotype of E151 is that of its LRs, which are short and delayed in their emergence. The atypical growth is seen as early as 3 DAP, though germination is not delayed. In E151, a zone where LR primordia are formed but where LRs do not emerge is quickly noticeable on the PR just below the cotyledons. Despite this LR-free zone which is maintained throughout life span, the mutant exhibits a root system denser than that of the WT. Even though this delay may affect the initiation of symbioses, the altered symbiotic phenotypes are not transient and symbiotic defects are seen throughout the mutant’s life.

E151 forms a few nodules but a high number of mycorrhizal structures. The explanation for this conundrum likely lies at the epidermis–cortex interface, a region previously considered as a checkpoint for micro-symbionts (Guinel and Geil, 2002). In E151, most of the ITs are arrested at this interface, but many fungal hyphae progress into the root cortex. Guinel and Geil (2002) proposed that rhizobial entry into the cortex is under ethylene control, and that excessive ethylene prohibits cortical penetration. The results obtained with E151 indicate the existence of yet another control because the E151 nodulation phenotype was not restored either with AVG or with Ag_2_SO_4_. Once in the cortex, both the IT and the fungal hypha meander, as if the pre-infection thread (PIT) and the pre-penetration apparatus (PPA) in the rhizobial and mycorrhizal symbiosis, respectively, did not form a proper blueprint. These two intracellular cytoskeletal structures are known to be responsible for the direction taken by the ITs and hyphae (Genre *et al.*, 2005; Fournier *et al.*, 2008).

### E151 shares phenotypic traits with hormone-disrupted plants

The LR characteristics of E151 are reminiscent of traits exhibited either by plants treated with CK or by transgenic plants affected in CK perception/homeostasis. Pea plants treated with BAP display fewer emerged LRs than control plants (Lorteau *et al.*, 2001). Furthermore, loss-of-function *Arabidopsis thaliana* CK receptor mutants (Riefler *et al.*, 2006), *M. truncatula* RNAi (RNA interference) plants for CK receptors (Gonzalez-Rizzo *et al.*, 2006), *M. truncatula* RNAi plants for the *LOG* gene (coding for the enzyme converting CKNTs to CK free bases; Mortier *et al.*, 2014), and *L. japonicus* transgenic roots overexpressing CK oxidase (CKX, the enzyme responsible for degrading CK; Lohar *et al.*, 2004) all exhibit higher LR number and LR density. Additionally, the nodulation traits of E151 bear similarities to those observed in plants with altered CK homeostasis or perception. Meandering ITs and stalled nodule primordia occur both in BAP-treated pea plants (Lorteau *et al.*, 2001) and in the *Ljhk1*/*Mtcre1* mutants (Gonzalez-Rizzo *et al.*, 2006; Murray *et al.*, 2007). Moreover, like those of E151, *Mtcre1* nodules are delayed in their development and are multilobed (Plet *et al.*, 2011). Yet, the *Mtcre1* mutant is different from E151 in that it does not exhibit an unusual mycorrhizal phenotype (Laffont *et al.*, 2015). The E151 mycorrhizal phenotype is, however, comparable with that of ABA-treated WT tomato (*Solanum lycopericum*) plants which display greater root colonization, mycorrhizal efficiency, and number of arbuscules than non-treated plants (Herrera-Medina *et al.*, 2007). Although no significant differences in ABA levels were found between the two pea lines, inoculated or not, the non-inoculated WT pea treated with ABA tended to phenocopy E151 in terms of LRs. However, this only occurred at the very high concentration of 10 μM ABA. Arguably, this level is not reflective of the physiological concentrations of a hormone. Moreover, it is in disagreement with the results of Liang and Harris (2005), who found that the legumes they tested increased their LR number upon ABA treatment.

### CK homeostasis is altered in the mutant E151

Here it is proposed that high CK levels are responsible for many of the E151 traits. It is hypothesized that the CK levels in the seed and/or growing PR are abnormal. As seedlings grow, high seed-based CK or high *de novo* root CK production would lead to high CK levels throughout most of the root system, altering seedling establishment. The LR primordia closer to the cotyledons would be more inhibited by higher CK levels than those more distant, resulting in an LR-free zone with non-emerged LR primordia. As the mutant PR grows, the CK levels would decrease and this would allow the acropetal development of numerous LRs to counterbalance the fewer and shorter LRs of the upper root system.

Upon inoculation of E151 with either micro-symbiont, CK levels drop to those similar to the WT, although this is less pronounced with rhizobia. A drop in CK with the rhizobia would be in agreement with the results of Clemow (2010) who measured, at 7 DAI, twice as many *PsCKX1* transcripts in nodulated roots as in non-nodulated roots of WT pea plants. An important distinction is that in Clemow’s study (2010), transcripts levels were measured in only those LRs that had been spot-inoculated, whereas here CK levels were measured from entire root systems. This difference in approach could explain why the CK drop could not be picked up in the nodulated WT because in entire roots, more subtle, local differences in CK levels could easily be missed. The results shown here emphasize the importance of temporal and spatial considerations when measuring hormone levels. It is worth noting that all the CK forms measured here are known substrates of CKX (Spíchal, 2012), and therefore could indeed be degraded by the enzyme. Young mycorrhizal roots had significantly lower CK levels than non-inoculated roots, suggesting that the presence of the fungal micro-symbiont has a severe effect on the endogenous CK levels of the plant. This is the first report in the literature of such an effect and it may be that the fungal micro-symbiont up-regulates *PsCKX* expression in a manner similar to *R. leguminosarum* as reported in Clemow (2010).

### CK homeostasis affects symbiosis development and functioning

The high CK levels in the young roots of E151 must have an impact on the symbioses because plants prepare in advance for micro-symbiont entry into the cortex. In E151, whereas rhizobia are usually stopped in their progression at the epidermal checkpoint, the fungi are stimulated and allowed to enter and spread extensively. Because nodulation requires the orchestration of two developmental programmes and mycorrhiza formation does not, the two micro-symbionts are not subjected to the same controls and it is proposed that they respond differently to the high CK levels in the whole root system. Whereas nodulation is known to require complex spatial and temporal CK dynamics (Ariel *et al.*, 2012), the results shown here would suggest that the pervasive high CK levels would allow mycorrhizal fungi to over-ride a cortical gate control.

As the micro-symbionts progress through the cortex, it is postulated that CK plays a role in the layout of the PITs and PPAs. Because the PIT formation has been likened to that of a pre-prophase band (Brewin, 2004), and because CK plays an essential role in the orientation of the plate deposition in LR founder cells (Moreira *et al.*, 2013), CK may have a regulatory function in the cytoskeletal re-organization leading to the PIT pattern. Because of the similarities between PPAs and PITs (Fournier *et al.*, 2008), it is suggested that CK has the same effect on PPAs as on PITs. Nevertheless, it cannot be excluded that other signals are at play. For example, E151 may have a problem perceiving reactive oxygen species (ROS) which are known to act as signals in both mutualistic symbioses (e.g. Arthikala *et al.*, 2014).

In nodulation, the IT is drawn towards the nodule founder cells. Held *et al.* (2014) proposed that a non-autonomous signal originating in the epidermis leads to inner cortical accumulation of CK. Once a specific, but still unknown, CK threshold is reached in the cortex, a nodule primordium is initiated. In a type of feedback loop, the nodule progenitor cell divisions result in epidermal CK accumulation which prevents further IT growth. In CK receptor mutants, lack of epidermal CK perception results in the hyperinfection of the root epidermis and in meandering cortical ITs (Held *et al.*, 2014). Accordingly, the high root CK levels of E151 would prevent a proper CK response because CK receptors would be activated before inoculation. Consequently, nodule primordia would be developmentally arrested, the PIT improperly positioned, and the IT twisting in the cortex.

In mycorrhiza formation, where no *de novo* structure hosts the micro-symbionts and therefore no organ founder cells are required, the fungal hypha does not require guidance in the cortex. In that tissue, the fungus grows in the apoplastic space and differentiates into numerous arbuscules and vesicles. The aberrant fungal growth in E151 must be a heavy tax on the plant because it requires the formation of a large amount of membrane to accommodate the fungus. Arbusculated cells are considered a strong photosynthate sink for the plant as they express high levels of sucrose synthase, the enzyme responsible for the hydrolysis of sucrose required for symbiotic transfer (Hohnjec *et al.*, 2003). Fungal sugar levels are tightly linked with ALP activity (Tisserant *et al.*, 1993; Ezawa *et al.*, 1999) and are related to PolyP accumulation which is indicative of an ineffective transfer of P from the fungus to the plant (Funamoto *et al.*, 2007). In E151, the symbiosis is impaired because fungal ALP activity and PolyP accumulation are decreased, suggesting that the AM fungus receives less sugar than in the WT and, as a consequence, that fungal P uptake is decreased. The symbiotic sink strength is probably exacerbated by the high root CK levels as CKs are known to increase sink strength (Werner *et al.*, 2008). It is thought that the high photosynthetic demand by the fungus may have obscured the results of the grafting experiments. Here, mycorrhiza formation was found to be controlled mostly by the shoot, whereas nodule organogenesis was clearly root controlled. This disparity does not match what is known in the literature (e.g. Resendes *et al.*, 2001) and may be explained by the physiological status of the plant. In E151, the arrested nodule primordia would not create a strong photosynthate sink whereas numerous arbuscules would.

## Conclusions

Nodulation mutants are useful tools to unravel the intricacy of both nodule and mycorrhizae development (e.g. Singh and Parniske, 2012), and pleiotropic mutants prove to be essential in defining the crucial role of plant hormones in regulating symbiotic development. In this work, E151 was characterized as a pleiotropic mutant assuming that its mutation is monogenic based on the work of Kneen *et al.* (1994). As such a mutant, E151 is altered in its responses to beneficial micro-symbionts and exhibits a root system architecture different from that of the WT. Furthermore, here, a positive role for CK in the mycorrhizal symbiosis has been demonstrated for the first time, although it had been previously thought that this hormone would be important in the initiation and the proper maintenance of the AM symbiosis (Ludwig-Müller, 2010). The results presented here support the recent work of Mortier *et al.* (2012) and of Sasaki *et al.* (2014), and highlight the key role CKs play in the intricacy of these two agriculturally important root symbioses.
